# Wind shear data at two different terrain types

**DOI:** 10.1016/j.dib.2019.104306

**Published:** 2019-07-24

**Authors:** Aliashim Albani, Mohd Zamri Ibrahim, Kim Hwang Yong

**Affiliations:** a Eastern Corridor Renewable Energy (ECRE), Universiti Malaysia Terengganu, 21030, Kuala Terengganu, Terengganu, Malaysia; b Faculty of Ocean Engineering Technology and Informatics, Universiti Malaysia Terengganu, 21030, Kuala Terengganu, Terengganu, Malaysia

**Keywords:** Wind shear, Wind energy, Extrapolation, Malaysia

## Abstract

The measurement of this data aims to evaluate the wind shear variability at three selected sites in Malaysia. The sites are Kudat in Sabah, Kijal in Terengganu and Langkawi in Kedah. Both sites in Kudat and Kijal is located in coastal areas with few buildings or trees, while the site in Langkawi is a coastal area with many buildings or trees. The variables were measured using the sensors that mounted on the wind mast with the maximum height from 55 m to 70 m from ground level. The variables measured were wind speed, wind direction, temperature, and pressure, while the wind shear data were directly generated using the power law equation. The averaged wind shear based on measured multiple height wind speed at selected sites is larger than the 1/7 law (0.143). Also, the value of wind shear was higher in order Langkawi > Kudat > Kijal. Ultimately, the wind shear data are essential and can be reused in the wind energy potential study, especially for data extrapolation to desired wind turbine hub height.

**Table:** Specifications Table

Subject	Energy
Specific subject area	Wind Energy
Type of data	Table, Graph
How data were acquired	Data measurement using the mechanical RNRG 40C model of anemometer, RNRG 200P model of wind vane, NRG 110S model of temperature sensor and NRG BP20 model of barometric pressure sensor that mounted on 30–70 m height wind mast
Data format	Analyzed, Filtered
Parameters for data collection	Wind shear, Wind speed, Wind direction, Pressure, and Temperature
Description of data collection	The data of wind speed, wind direction, temperature, and pressure were measured using the NRG System equipment that mounted on the wind mast. The mounting of these anemometers follows the IEC 61400-12-1 [1] as well as a recommendation in the IEA guidelines [2]. The wind shear generated by using a power law equation based on two different heights of collected wind speed.
Data source location	Kudat, Sabah, Malaysia (7°1′45.33″ N, 116°44′47.98″ E) Kijal, Terengganu, Malaysia (4°20′50.70″ N, 103°28′34.74″ E) Langkawi, Kedah, Malaysia (6°21′37.92″ N, 99°41′16.62″ E)
Data accessibility	Data are included in this article

**Table:** 

**Value of the Data** • The dataset acquired in this study reveals the diurnal and monthly variability of wind shear at three selected sites in Malaysia with different terrain types. • The data measurement was following the IEC 61400-12-1 as well as a recommendation in the IEA guidelines. • The dataset will provide valuable information for wind data extrapolation in energy exploration purposes. • No detailed wind shear data are published anywhere. Hence, this paper will benefit researchers and other stakeholders from various fields, especially wind energy. • The information composed in this article can be used as a basis for determining the wind energy potential in Malaysia as two out of three selected sites; Kudat and Kijal showed the promising data for wind energy development.

## 1 Data

**Fig. 1:**
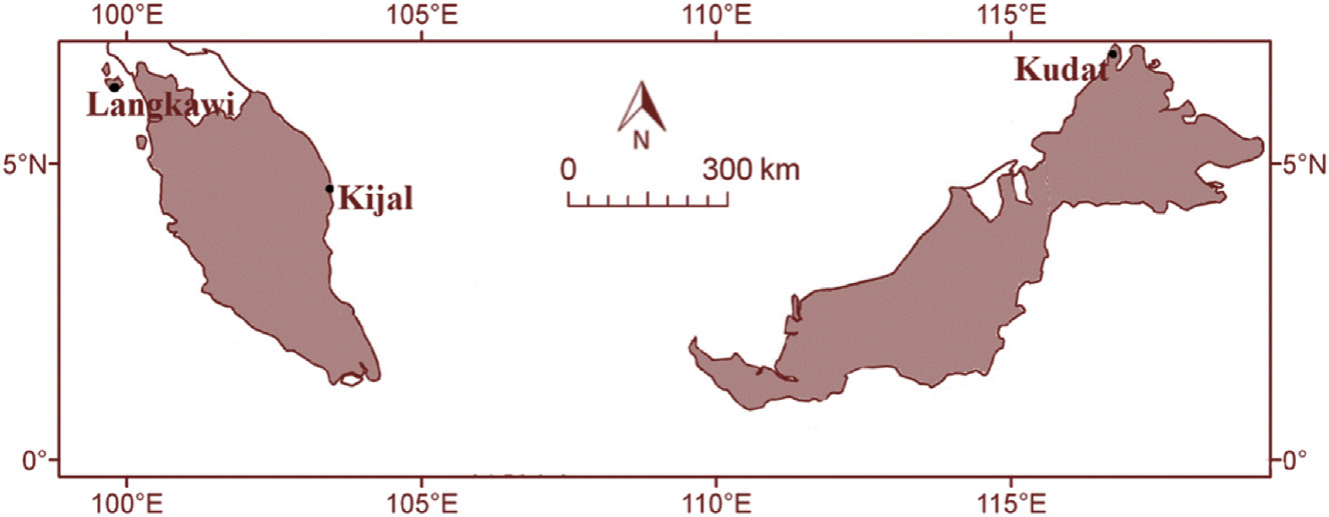
Map showing the selected sites.

**Fig. 2:**
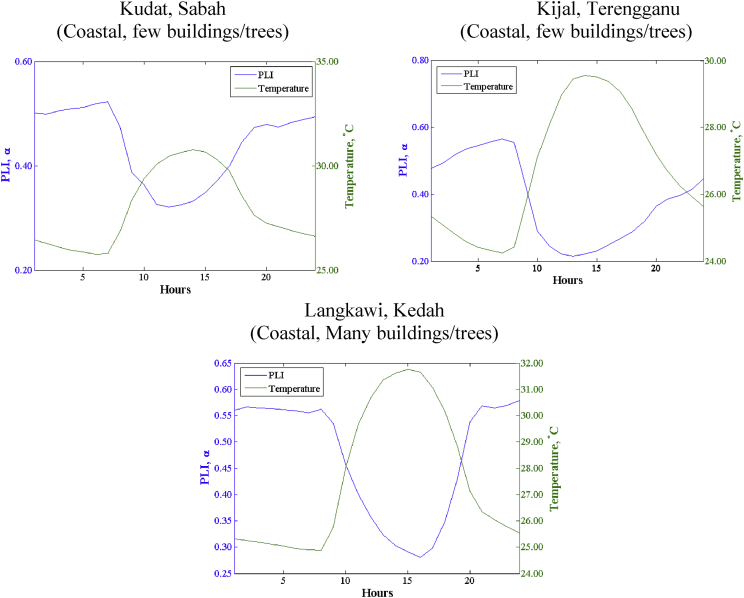
The Diurnal Variation of wind shear at Different Sites.

Four variables were measured using the different sensors, which mounted on the same mast tower. Some wind masts have two or three variables, as not all of them have all four variables. However, all measurement masts should have important variable wind speed and direction. Also, essential data, namely as wind shear, was directly computed using the power law equation (See Eq. (1)). The description of filtered and analyzed data was presented in Table 1, Table 2, Table 3, Table 4, Table 5, and Fig. 1, Fig. 2. The following are measured and computed variables:

a. Wind Speed

**Table 1:** The coordinates, heights of the wind masts, and the period of data for every selected site.

Station Sites and Coordinates	Data Parameters, Heights and Accuracies	Measurement Periods, Number of Data and Data Recovery	Sites Descriptions
Kudat (7°1′45.33″ N, 116°44′47.98″ E)	Wind speed, 10 m, (±0.4 m/s)	May 2014–April 2015 (12 Months) Recovery: 99%	**Coastal, few buildings/trees** Located at a site facing the ocean wind from West (W) to South (S) direction. Few trees observed on the North (N) and the East (E), where the surface wind speed was predominantly blowing.
	Wind speed, 35 m, (±0.4 m/s)		
	Wind speed, 50 m, (±0.4 m/s)		
	Wind speed, 70 m, (±0.4 m/s)		
	Wind direction, 10 m, (±1°)		
	Wind direction, 70 m, (±1°)		
	Temperature, 10 m, (±0.5 °C)		
	Pressure, 10 m, (±0.5 mbar)		
Kijal (4°20′50.70″ N, 103°28′34.74″ E)	Wind speed, 10 m, (±0.4 m/s)	May 2013–April 2014 (12 Months) Recovery: 99%	**Coastal, few buildings/trees** Located at a site facing the ocean wind from North (N) to East (E) direction. However, a few trees and buildings observed on the South (S) and the West (W), where the surface wind speed was predominantly blowing.
	Wind speed, 15 m, (±0.4 m/s)		
	Wind speed, 40 m, (±0.4 m/s)		
	Wind speed, 55 m, (±0.4 m/s)		
	Wind direction, 10 m, (±1°)		
	Wind direction, 55 m, (±1°)		
	Temperature, 10 m, (±0.5 °C)		
	Pressure, 10 m, (±0.5 mbar)		
Langkawi 6°21′37.92″ N, 99°41′16.62″ E	Wind speed 10 m (±0.4 m/s)	May 2014–April 2015 (12 Months) Recovery: 99%	**Coastal, Many buildings/trees** Located at a site facing the ocean wind from West (W) to North (N) direction. Many trees observed on the East (E) and the South (S), where the surface wind speed was predominantly blowing.
	Wind speed 30 m (±0.4 m/s)		
	Wind speed 40 m (±0.4 m/s)		
	Wind speed 70 m (±0.4 m/s)		
	Wind direction 10 m (±1°)		
	Wind direction 70 m (±1°)		
	Temperature 10 m (±0.5 °C)		
	Pressure 10 m (±0.5 mbar)

**Table 2:** The statistical analysis of measured wind data.

Sites	Kudat	Kijal	Langkawi
Height of wind mast	10 m, 35 m, 50 m, and 70 m	10 m, 15 m, 40 m, and 55 m	10 m, 30 m, 40 m, and 70 m
Averaged Temperature	27.00 °C	27.10 °C	28.20 °C
Pressure	1007.00 mbar	987.40 mbar	990.00 mbar
Air density	1.1688 kg/m^3^	1.1661 kg/m^3^	1.1445 kg/m^3^
Scale parameter, c	10 m: 3.20 m/s	10 m: 2.77 m/s	10 m: 1.63 m/s
	35 m: 5.26 m/s	15 m: 3.44 m/s	30 m: 2.97 m/s
	50 m: 6.08 m/s	40 m: 3.81 m/s	40 m: 3.60 m/s
	70 m: 6.67 m/s	55 m: 4.57 m/s	70 m: 4.47 m/s
Shape parameter, k	10 m: 1.84	10 m: 1.76	10 m: 1.63
	35 m: 2.02	15 m: 1.78	30 m: 1.76
	50 m: 2.19	40 m: 1.81	40 m: 1.69
	70 m: 2.25	55 m: 1.78	70 m: 1.70
Averaged wind speed	10 m: 2.72 m/s	10 m: 2.31 m/s	10 m: 1.46 m/s
	35 m: 4.60 m/s	15 m: 2.92 m/s	30 m: 2.64 m/s
	50 m: 5.32 m/s	40 m: 3.37 m/s	40 m: 3.22 m/s
	70 m: 5.86 m/s	55 m: 4.02 m/s	70 m: 3.81 m/s
Wind power density	10 m: 11.76 W/m^2^	10 m: 7.19 W/m^2^	10 m: 1.78 W/m^2^
	35 m: 56.88 W/m^2^	15 m: 14.52 W/m^2^	30 m: 10.53 W/m^2^
	50 m: 87.99 W/m^2^	40 m: 22.31 W/m^2^	40 m: 19.11 W/m^2^
	70 m: 117.60 W/m^2^	55 m: 37.88 W/m^2^	70 m: 31.65 W/m^2^

**Table 3:** The diurnal and monthly wind shear in Kudat, Sabah (Coastal, few buildings/trees).

Diurnal	10m/35m	10m/50m	10m/70m	35m/50m	35m/70m	50m/70m
00	0.46 ± 0.03	0.45 ± 0.03	0.42 ± 0.01	0.41 ± 0.01	0.36 ± 0.02	0.31 ± 0.06
01	0.44 ± 0.01	0.44 ± 0.01	0.42 ± 0.02	0.42 ± 0.02	0.39 ± 0.04	0.36 ± 0.02
02	0.45 ± 0.02	0.45 ± 0.01	0.43 ± 0.02	0.43 ± 0.03	0.39 ± 0.04	0.34 ± 0.08
03	0.45 ± 0.03	0.45 ± 0.03	0.43 ± 0.02	0.45 ± 0.02	0.39 ± 0.03	0.32 ± 0.04
04	0.45 ± 0.02	0.45 ± 0.02	0.43 ± 0.02	0.44 ± 0.07	0.39 ± 0.01	0.33 ± 0.07
05	0.46 ± 0.02	0.46 ± 0.02	0.44 ± 0.02	0.44 ± 0.01	0.40 ± 0.02	0.35 ± 0.05
06	0.48 ± 0.02	0.47 ± 0.02	0.45 ± 0.02	0.44 ± 0.01	0.40 ± 0.02	0.36 ± 0.02
07	0.47 ± 0.02	0.47 ± 0.02	0.45 ± 0.02	0.45 ± 0.11	0.42 ± 0.05	0.40 ± 0.11
08	0.38 ± 0.01	0.39 ± 0.01	0.38 ± 0.01	0.43 ± 0.01	0.37 ± 0.01	0.30 ± 0.01
09	0.35 ± 0.01	0.34 ± 0.01	0.33 ± 0.01	0.33 ± 0.04	0.29 ± 0.01	0.25 ± 0.08
10	0.33 ± 0.02	0.32 ± 0.02	0.30 ± 0.01	0.30 ± 0.02	0.26 ± 0.01	0.21 ± 0.01
11	0.32 ± 0.05	0.31 ± 0.03	0.29 ± 0.02	0.28 ± 0.01	0.23 ± 0.03	0.19 ± 0.09
12	0.32 ± 0.02	0.31 ± 0.01	0.29 ± 0.02	0.27 ± 0.11	0.23 ± 0.03	0.19 ± 0.17
13	0.32 ± 0.02	0.31 ± 0.02	0.29 ± 0.01	0.28 ± 0.04	0.23 ± 0.01	0.17 ± 0.01
14	0.34 ± 0.01	0.33 ± 0.01	0.30 ± 0.01	0.29 ± 0.02	0.24 ± 0.03	0.19 ± 0.05
15	0.36 ± 0.02	0.35 ± 0.02	0.32 ± 0.02	0.29 ± 0.01	0.25 ± 0.02	0.21 ± 0.07
16	0.39 ± 0.02	0.38 ± 0.01	0.35 ± 0.03	0.33 ± 0.06	0.29 ± 0.04	0.24 ± 0.09
17	0.41 ± 0.01	0.40 ± 0.01	0.37 ± 0.02	0.36 ± 0.04	0.31 ± 0.01	0.25 ± 0.02
18	0.43 ± 0.01	0.43 ± 0.01	0.40 ± 0.01	0.41 ± 0.05	0.35 ± 0.03	0.28 ± 0.01
19	0.43 ± 0.01	0.43 ± 0.01	0.40 ± 0.01	0.42 ± 0.01	0.35 ± 0.03	0.28 ± 0.07
20	0.44 ± 0.01	0.43 ± 0.02	0.40 ± 0.02	0.39 ± 0.11	0.34 ± 0.04	0.29 ± 0.04
21	0.44 ± 0.01	0.43 ± 0.01	0.41 ± 0.01	0.43 ± 0.01	0.37 ± 0.01	0.30 ± 0.08
22	0.44 ± 0.01	0.43 ± 0.01	0.41 ± 0.01	0.41 ± 0.04	0.36 ± 0.01	0.30 ± 0.04
23	0.44 ± 0.01	0.44 ± 0.02	0.41 ± 0.01	0.43 ± 0.04	0.36 ± 0.01	0.28 ± 0.10
Monthly	10m/35m	10m/50m	10m/70m	35m/50m	35m/70m	50m/70m
January	0.46 ± 0.01	0.47 ± 0.01	0.47 ± 0.01	0.48 ± 0.02	0.47 ± 0.01	0.46 ± 0.03
February	0.44 ± 0.02	0.45 ± 0.02	0.44 ± 0.01	0.47 ± 0.11	0.43 ± 0.04	0.39 ± 0.01
March	0.41 ± 0.01	0.43 ± 0.01	0.42 ± 0.01	0.48 ± 0.01	0.43 ± 0.04	0.37 ± 0.10
April	0.41 ± 0.01	0.41 ± 0.01	0.40 ± 0.01	0.42 ± 0.06	0.37 ± 0.04	0.32 ± 0.02
May	0.41 ± 0.01	0.41 ± 0.01	0.38 ± 0.01	0.40 ± 0.06	0.34 ± 0.01	0.27 ± 0.05
June	0.37 ± 0.01	0.35 ± 0.01	0.32 ± 0.01	0.27 ± 0.04	0.22 ± 0.02	0.15 ± 0.03
July	0.36 ± 0.01	0.34 ± 0.01	0.30 ± 0.01	0.26 ± 0.01	0.20 ± 0.01	0.14 ± 0.07
August	0.39 ± 0.02	0.36 ± 0.02	0.32 ± 0.01	0.26 ± 0.03	0.21 ± 0.01	0.15 ± 0.01
September	0.40 ± 0.02	0.37 ± 0.01	0.34 ± 0.01	0.26 ± 0.02	0.22 ± 0.02	0.18 ± 0.07
October	0.38 ± 0.02	0.36 ± 0.01	0.32 ± 0.01	0.29 ± 0.01	0.23 ± 0.01	0.17 ± 0.01
November	0.40 ± 0.02	0.42 ± 0.02	0.41 ± 0.01	0.50 ± 0.02	0.42 ± 0.01	0.34 ± 0.02
December	0.42 ± 0.02	0.43 ± 0.01	0.42 ± 0.01	0.48 ± 0.01	0.42 ± 0.01	0.35 ± 0.01

**Table 4:** The diurnal and monthly wind shear in Kijal, Terengganu (Coastal, few buildings/trees).

Diurnal	10m/15m	10m/40m	10m/55m	15m/40m	15m/55m	40m/55m
00	0.63 ± 0.30	0.33 ± 0.09	0.27 ± 0.09	0.20 ± 0.10	0.28 ± 0.09	0.51 ± 0.33
01	0.84 ± 0.32	0.38 ± 0.08	0.31 ± 0.06	0.18 ± 0.12	0.26 ± 0.07	0.51 ± 0.25
02	0.85 ± 0.27	0.39 ± 0.12	0.32 ± 0.08	0.20 ± 0.13	0.29 ± 0.08	0.56 ± 0.44
03	0.91 ± 0.24	0.42 ± 0.10	0.34 ± 0.08	0.22 ± 0.11	0.31 ± 0.08	0.57 ± 0.40
04	0.92 ± 0.35	0.45 ± 0.10	0.36 ± 0.09	0.25 ± 0.11	0.32 ± 0.09	0.55 ± 0.43
05	0.97 ± 0.38	0.46 ± 0.10	0.37 ± 0.09	0.25 ± 0.08	0.32 ± 0.08	0.54 ± 0.38
06	0.95 ± 0.23	0.44 ± 0.11	0.36 ± 0.07	0.23 ± 0.13	0.31 ± 0.08	0.55 ± 0.48
07	0.96 ± 0.28	0.46 ± 0.08	0.37 ± 0.07	0.25 ± 0.09	0.32 ± 0.08	0.52 ± 0.36
08	0.83 ± 0.30	0.44 ± 0.08	0.36 ± 0.07	0.27 ± 0.09	0.33 ± 0.06	0.49 ± 0.36
09	0.55 ± 0.27	0.28 ± 0.10	0.23 ± 0.08	0.16 ± 0.10	0.23 ± 0.08	0.44 ± 0.30
10	0.41 ± 0.23	0.18 ± 0.08	0.15 ± 0.06	0.09 ± 0.01	0.19 ± 0.07	0.49 ± 0.37
11	0.34 ± 0.17	0.14 ± 0.07	0.11 ± 0.05	0.05 ± 0.01	0.18 ± 0.07	0.58 ± 0.34
12	0.33 ± 0.12	0.12 ± 0.07	0.10 ± 0.04	0.04 ± 0.01	0.18 ± 0.03	0.60 ± 0.35
13	0.33 ± 0.29	0.13 ± 0.09	0.11 ± 0.07	0.05 ± 0.01	0.18 ± 0.07	0.59 ± 0.43
14	0.34 ± 0.14	0.14 ± 0.05	0.11 ± 0.06	0.05 ± 0.05	0.19 ± 0.07	0.60 ± 0.29
15	0.37 ± 0.09	0.15 ± 0.04	0.12 ± 0.04	0.06 ± 0.05	0.20 ± 0.05	0.63 ± 0.20
16	0.41 ± 0.25	0.19 ± 0.05	0.15 ± 0.05	0.10 ± 0.11	0.22 ± 0.07	0.60 ± 0.30
17	0.44 ± 0.07	0.21 ± 0.04	0.17 ± 0.04	0.11 ± 0.06	0.23 ± 0.04	0.62 ± 0.31
18	0.52 ± 0.18	0.25 ± 0.09	0.20 ± 0.05	0.14 ± 0.12	0.25 ± 0.06	0.58 ± 0.42
19	0.57 ± 0.23	0.27 ± 0.06	0.22 ± 0.05	0.15 ± 0.09	0.26 ± 0.07	0.58 ± 0.35
20	0.59 ± 0.25	0.30 ± 0.07	0.24 ± 0.07	0.17 ± 0.10	0.26 ± 0.08	0.53 ± 0.31
21	0.65 ± 0.26	0.31 ± 0.09	0.25 ± 0.04	0.16 ± 0.12	0.25 ± 0.09	0.51 ± 0.35
22	0.68 ± 0.25	0.32 ± 0.07	0.26 ± 0.07	0.17 ± 0.11	0.24 ± 0.07	0.47 ± 0.36
23	0.69 ± 0.26	0.33 ± 0.08	0.27 ± 0.05	0.18 ± 0.14	0.27 ± 0.07	0.57 ± 0.47
Monthly	10m/15m	10m/40m	10m/55m	15m/40m	15m/55m	40m/55m
January	0.67 ± 0.12	0.25 ± 0.03	0.32 ± 0.02	0.08 ± 0.04	0.21 ± 0.03	0.61 ± 0.01
February	0.58 ± 0.17	0.26 ± 0.04	0.33 ± 0.05	0.13 ± 0.06	0.25 ± 0.05	0.60 ± 0.23
March	0.59 ± 0.19	0.29 ± 0.06	0.35 ± 0.05	0.17 ± 0.07	0.28 ± 0.05	0.59 ± 0.20
April	0.67 ± 0.17	0.32 ± 0.06	0.37 ± 0.05	0.17 ± 0.08	0.27 ± 0.07	0.58 ± 0.38
May	0.12 ± 0.23	0.20 ± 0.07	0.30 ± 0.04	0.23 ± 0.09	0.36 ± 0.06	0.76 ± 0.35
June	0.10 ± 0.22	0.22 ± 0.05	0.25 ± 0.05	0.27 ± 0.09	0.30 ± 0.06	0.39 ± 0.17
July	0.56 ± 0.16	0.25 ± 0.04	0.29 ± 0.04	0.13 ± 0.07	0.21 ± 0.05	0.47 ± 0.16
August	0.62 ± 0.20	0.24 ± 0.05	0.29 ± 0.04	0.08 ± 0.05	0.19 ± 0.04	0.55 ± 0.13
September	0.68 ± 0.21	0.25 ± 0.07	0.30 ± 0.06	0.07 ± 0.08	0.18 ± 0.05	0.53 ± 0.15
October	0.60 ± 0.21	0.28 ± 0.07	0.30 ± 0.05	0.15 ± 0.08	0.21 ± 0.06	0.42 ± 0.19
November	0.74 ± 0.20	0.34 ± 0.07	0.36 ± 0.05	0.17 ± 0.08	0.24 ± 0.07	0.45 ± 0.17
December	0.70 ± 0.24	0.33 ± 0.07	0.37 ± 0.05	0.17 ± 0.09	0.27 ± 0.07	0.58 ± 0.16

**Table 5:** The diurnal and monthly wind shear in Langkawi, Kedah (Coastal, Many buildings/trees).

Diurnal		10m/30m	10m/40m	10m/70m	30m/40m	30m/70m	40m/70m
00		0.71 ± 0.15	0.75 ± 0.10	0.65 ± 0.08	0.92 ± 0.60	0.58 ± 0.17	0.40 ± 0.30
01		0.68 ± 0.18	0.73 ± 0.11	0.62 ± 0.09	0.89 ± 0.61	0.53 ± 0.22	0.35 ± 0.40
02		0.72 ± 0.12	0.76 ± 0.16	0.65 ± 0.09	0.90 ± 0.65	0.55 ± 0.16	0.37 ± 0.35
03		0.75 ± 0.13	0.78 ± 0.11	0.66 ± 0.08	0.88 ± 0.66	0.55 ± 0.19	0.38 ± 0.26
04		0.72 ± 0.16	0.71 ± 0.12	0.65 ± 0.08	0.69 ± 0.60	0.55 ± 0.20	0.48 ± 0.25
05		0.72 ± 0.14	0.75 ± 0.13	0.64 ± 0.07	0.88 ± 0.63	0.54 ± 0.13	0.36 ± 0.30
06		0.68 ± 0.14	0.72 ± 0.12	0.61 ± 0.06	0.88 ± 0.48	0.52 ± 0.17	0.33 ± 0.27
07		0.72 ± 0.15	0.75 ± 0.15	0.64 ± 0.10	0.85 ± 0.63	0.53 ± 0.17	0.37 ± 0.26
08		0.69 ± 0.12	0.72 ± 0.13	0.62 ± 0.06	0.86 ± 0.64	0.53 ± 0.18	0.36 ± 0.27
09		0.71 ± 0.15	0.70 ± 0.13	0.61 ± 0.09	0.66 ± 0.43	0.49 ± 0.15	0.40 ± 0.21
10		0.68 ± 0.09	0.68 ± 0.08	0.58 ± 0.04	0.69 ± 0.31	0.46 ± 0.11	0.34 ± 0.18
11		0.62 ± 0.06	0.61 ± 0.07	0.51 ± 0.03	0.61 ± 0.39	0.37 ± 0.10	0.24 ± 0.20
12		0.52 ± 0.06	0.53 ± 0.05	0.44 ± 0.04	0.55 ± 0.26	0.35 ± 0.11	0.24 ± 0.18
13		0.48 ± 0.09	0.49 ± 0.06	0.41 ± 0.04	0.56 ± 0.23	0.32 ± 0.08	0.20 ± 0.15
14		0.47 ± 0.08	0.49 ± 0.07	0.41 ± 0.05	0.56 ± 0.30	0.33 ± 0.09	0.21 ± 0.11
15		0.44 ± 0.08	0.46 ± 0.06	0.39 ± 0.04	0.52 ± 0.16	0.32 ± 0.05	0.22 ± 0.07
16		0.42 ± 0.05	0.46 ± 0.05	0.40 ± 0.04	0.64 ± 0.19	0.37 ± 0.06	0.23 ± 0.09
17		0.40 ± 0.14	0.43 ± 0.09	0.38 ± 0.07	0.55 ± 0.29	0.36 ± 0.09	0.27 ± 0.11
18		0.42 ± 0.09	0.49 ± 0.07	0.43 ± 0.05	0.75 ± 0.39	0.43 ± 0.13	0.27 ± 0.19
19		0.56 ± 0.08	0.60 ± 0.06	0.52 ± 0.05	0.74 ± 0.35	0.47 ± 0.17	0.33 ± 0.24
20		0.62 ± 0.14	0.66 ± 0.10	0.59 ± 0.08	0.78 ± 0.33	0.54 ± 0.15	0.41 ± 0.22
21		0.72 ± 0.11	0.73 ± 0.08	0.63 ± 0.08	0.77 ± 0.40	0.50 ± 0.18	0.37 ± 0.22
22		0.71 ± 0.10	0.76 ± 0.08	0.63 ± 0.06	0.96 ± 0.45	0.53 ± 0.17	0.31 ± 0.26
23		0.74 ± 0.17	0.75 ± 0.14	0.66 ± 0.10	0.79 ± 0.66	0.56 ± 0.22	0.44 ± 0.45
Monthly		10m/30m	10m/40m	10m/70m	30m/40m	30m/70m	40m/70m
January		0.67 ± 0.07	0.68 ± 0.05	0.59 ± 0.04	0.71 ± 0.19	0.48 ± 0.09	0.36 ± 0.07
February		0.62 ± 0.05	0.64 ± 0.05	0.56 ± 0.03	0.72 ± 0.23	0.48 ± 0.11	0.35 ± 0.11
March		0.57 ± 0.06	0.59 ± 0.06	0.51 ± 0.04	0.64 ± 0.32	0.42 ± 0.09	0.31 ± 0.17
April		0.57 ± 0.11	0.59 ± 0.10	0.52 ± 0.05	0.65 ± 0.40	0.45 ± 0.13	0.34 ± 0.20
May		0.55 ± 0.12	0.58 ± 0.10	0.50 ± 0.06	0.69 ± 0.40	0.43 ± 0.14	0.30 ± 0.20
June		0.45 ± 0.14	0.60 ± 0.08	0.50 ± 0.07	1.19 ± 0.52	0.57 ± 0.15	0.26 ± 0.20
July		0.53 ± 0.11	0.57 ± 0.08	0.48 ± 0.06	0.70 ± 0.37	0.42 ± 0.11	0.28 ± 0.16
August		0.55 ± 0.12	0.57 ± 0.12	0.51 ± 0.07	0.68 ± 0.40	0.46 ± 0.12	0.35 ± 0.19
September		0.57 ± 0.12	0.61 ± 0.08	0.51 ± 0.07	0.77 ± 0.43	0.44 ± 0.15	0.27 ± 0.20
October		0.53 ± 0.11	0.60 ± 0.09	0.49 ± 0.06	0.85 ± 0.50	0.45 ± 0.15	0.24 ± 0.20
November		0.66 ± 0.10	0.66 ± 0.08	0.57 ± 0.06	0.66 ± 0.33	0.45 ± 0.11	0.34 ± 0.15
December		0.74 ± 0.08	0.73 ± 0.08	0.63 ± 0.04	0.70 ± 0.27	0.48 ± 0.09	0.37 ± 0.19

The wind speed is measured using the mechanical RNRG 40C model of anemometer, which commercially designed with three conical-shaped cups mounted on short arms connected to a rotating vertical shaft. The three-cup anemometer chosen as this design has a more uniform torque throughout a revolution. The anemometer rotates by the wind and generates a signal proportional to wind speed in meter per second.

b. Wind Direction

Wind direction defined as the direction from which the wind speed is coming from, and it is measured in degrees clockwise from true north. It is measured by an RNRG 200P model of wind vane, which consists of a tail attached to one end of a horizontal shaft.

c. Temperature

Ambient temperature is measured using the NRG 110S model of the temperature sensor. The temperature data were used to calculate air density.

d. Pressure

Absolute pressure is measured using the NRG BP20 model of the barometric pressure sensor. The pressure data were used to calculate air density.

e. Wind shear

The wind shear, α is generated by direct computation using the power law equation of two different heights of measured wind data.

## 2 Experimental design, materials, and methods

The selected sites are Kudat in Sabah, Kijal in Terengganu and Langkawi in Kedah, as shown in Fig. 1. The sites were selected based on field inspection trips and preliminary wind resource maps, which are developed using meteorological wind data. The selection of sites also considers the difficulties of access, the availability of grid connection, and the wind quality. At least one additional anemometer should be mounted on the tower to generate the wind shear. The mounting of these additional anemometer follows the IEC 61400-12-1 [1], as well as a recommendation in the IEA guidelines [2]. The maximum height of wind mast tower is different for every site, ranging from 55 m to 70 m (m.a.g.l). Higher measurement heights will reduce the uncertainty of the vertical extrapolation of the wind speed. The wind data were measured using the NRG System equipment, which is a technology from the United States. The station sites, together with coordinates and site description, are shown in Table 1. The data were measured at least one year in the period to get the data trend during different monsoonal seasons; (i) Northeast Monsoon (October to March), and (ii) Southwest Monsoon (May to September).

The quality of the measured data was assured by data screening and validation using the Meteo module in WindPRO program [3]. Generally, the procedures of data screening and validation include comparisons of measured values to upper and lower limits; these limits may be the instrument threshold or local atmospheric historical data. As an example, it is impossible to obtain a negative value of temperature in Malaysia as it is located in the equator line. Thus, those invalid data would be considered to be eliminated from the datasets. The screening is an iterative process in which range checks and other screening criteria are revised based on experience. The criteria of data that would be eliminated are listed as the following [4]:

a. The wind speed value is less than zero or greater than 25 m/s.
b. The wind direction is less than zero or greater than 360°.
c. The temperature value is greater than the local record high, which is the local maximum temperature of 35 °C and less than the local record low, which is the local minimum temperature of 10 °C.
d. The pressure value is greater than 1060 mbar (sea level) and less than 940 mbar (sea level).

The data are checked and validated every month to detect possible defects in time and to limit any data losses. The data validation was employed based on methods suggested by Refs. [5], [6]. The missing data and outliers would affect the result of wind resource assessment [7]. The statistical analysis of measured data was presented in Table 2. The data analysis and plotting were conducted in MATLAB program.

The Hellmann Power Law is the most frequent model to determine the wind shear, which is used in several wind shear studies [8], [9], [10], [11], [12], [13]. The following is the Hellmann Power Law to determine wind shear, *α*:

(1) $$\alpha =\frac{\text{In}\left(\frac{v_{2}}{v_{1}}\right)}{\text{In}\left(\frac{z_{1}}{z_{2}}\right)}$$

The extrapolation of wind speed data measured at a certain height to desired hub height of wind turbine is a point of interest to many wind energy applications. The extrapolation of wind speed data to different heights varies considerably, depending on whether the extrapolation is conducted over complex or relatively flat terrain type. To do extrapolation, the proper estimation of wind shears was needed to reduce the inaccuracy of extrapolation results. The wind shear is the variation of wind speed with elevation. It is generally accepted that as terrain complexities (ruggedness) increase, the value of wind shear also increases. As presented by Ref. [13], the average wind shear for open and flat terrain type is up to 0.19, for few trees or buildings (0.24–0.43) and many trees or buildings (0.48 and above). The wind shear that generated by direct computation using wind data at two different heights were presented in Table 3 to Table 5 and Fig. 2. The diurnal and monthly wind shear were analyzed and found that the value is too large or negative. This phenomenon occurred because wind speed data at each altitude is variable, sometimes data at high altitude is lower than low altitudes. Accordingly, the best approach to determine wind shear was by averaging all of the value, which is also known as the overall mean averaging method [14]. The averaged wind shear for Kudat, Kijal and Langkawi are 0.38, 0.25 and 0.48 respectively. The averaged wind shear based on measured multiple height wind speed is larger than the 1/7 Law (0.143). The 1/7 Law (0.143) was introduced by Frost in 1974, and is the standard value used for data extrapolation for single height anemometer. It is a common practice among Malaysian researchers to use 1/7 Law (0.143) as the wind shear to determine the value of wind speed for the desired heights [15], [16], [17], [18], [19]. This practice leads to the inaccuracy of result data.
